# BrainWave Nets: Are Sparse Dynamic Models Susceptible to Brain Manipulation Experimentation?

**DOI:** 10.3389/fnsys.2020.527757

**Published:** 2020-11-26

**Authors:** Diego C. Nascimento, Marco A. Pinto-Orellana, Joao P. Leite, Dylan J. Edwards, Francisco Louzada, Taiza E. G. Santos

**Affiliations:** ^1^Institute of Mathematical Science and Computing, University of São Paulo, Sao Carlos, Brazil; ^2^Departamento de Matemática, Universidad de Atacama de Chile, Copiapo, Chile; ^3^Institutt for maskinelektronikk og kjemi, Oslo Metropolitan University, Oslo, Norway; ^4^Ribeirao Preto Medical School, University of São Paulo, Ribeirao Preto, Brazil; ^5^Moss Rehabilitation Research Institute, Elkins Park, PA, United States; ^6^School of Medical and Health Sciences, Edith Cowan University, Joondalup, WA, Australia

**Keywords:** state space models, multilayer networks, high-dimensional time series model, transcranial direct current stimulation, dynamic graphical model

## Abstract

Sparse time series models have shown promise in estimating contemporaneous and ongoing brain connectivity. This paper was motivated by a neuroscience experiment using EEG signals as the outcome of our established interventional protocol, a new method in neurorehabilitation toward developing a treatment for visual verticality disorder in post-stroke patients. To analyze the [complex outcome measure (EEG)] that reflects neural-network functioning and processing in more specific ways regarding traditional analyses, we make a comparison among sparse time series models (classic VAR, GLASSO, TSCGM, and TSCGM-modified with non-linear and iterative optimizations) combined with a graphical approach, such as a Dynamic Chain Graph Model (DCGM). These dynamic graphical models were useful in assessing the role of estimating the brain network structure and describing its causal relationship. In addition, the class of DCGM was able to visualize and compare experimental conditions and brain frequency domains [using finite impulse response (FIR) filter]. Moreover, using multilayer networks, the results corroborate with the susceptibility of sparse dynamic models, bypassing the false positives problem in estimation algorithms. We conclude that applying sparse dynamic models to EEG data may be useful for describing intervention-relocated changes in brain connectivity.

## 1. Introduction

In the area of neuroscience, work related to the brain network structure, as well as its dynamics, has increased due to technological developments (high resolution and storage capacity). Notwithstanding, the field aims to understand “how” and “why” the effects/events occur based on learning probabilistic connection structures to assume some feasible causal inference (Pearl, 2014). There is thus an immediate urge to map its complex organization, and two types of connectivity are commonly studied: functional and dynamic. Functional connectivity is a statistical measure of the correlation within observations in the same time-lapse, and dynamic connectivity is the relationship among the measurements compared with their previous value impact.

Thus, the links among anatomical parcellations of the brain are described by their similarity patterns; for instance, a channel represents the activity of a group of neurons, and it is measured according to its space relation, time, and frequency domains. Statistical significance tests are often conducted to estimate the existence of those links in order to project an estimated topology regarding the interaction among this observed group of neurons. For example, brain dynamics are measured as biosignals through an electroencephalogram (EEG), functional magnetic resonance imaging (fMRI), diffusion tensor imaging (DTI), and Doppler ultrasound. Most recently, effective brain network connectivity changes following non-invasive transcranial stimulation has been investigated using fMRI (Fiori et al., 2018), fNIRS (Cao et al., 2018), and EEG (Baxter et al., 2017).

Biosignals are often presented as time-indexed values in which their modeling requires components that may also vary over time; the dynamic factor models, together with graphical representation, can help this demand. Time-varying Bayesian dynamic models were introduced, and variations were then developed, such as the Gaussian graphical model and usage of splines (for further details, please see Quintana and West, 1987; Queen and Smith, 1993; Carvalho et al., 2007; Anacleto et al., 2017). Nevertheless, this approach is always suitable for multivariate series whose component univariate series are similar and share a common structure.

Network modeling is a mathematical framework, part of graph theory, used to represent and analyze relationships in multivariate data. Recent advances in network estimation have moved the emphasis of the analysis from single-layer networks to multilayer structures facilitating the interpretation of multivariate relationships (Kivelä et al., 2014). This paradigm shift expands the possibilities of extracting information about complex systems, and conducts a multilayer network estimation of biosignals that can incorporate the change in time and/or different frequencies.

Multilayer analysis can reveal the complexity of the human brain, and investigations can thus show effective functional roles in brain region activation and visual representation (De Domenico, 2017; Gratton et al., 2018). In this context, two main approaches are often seen, multimodal connectivity or structural-functional relationships (different layers represent replicated nodes and their interaction) and time-varying networks (evolution of the temporal snapshots).

The concept of sparse multivariate time series with multiplex networks benefits the analysis of brain dynamic activation by using the frequency-domain approaches as physiologically applicable biosignal denoising. Decomposition methods in the frequency domain are generally used in conjunction with graphical models; for example, Bach and Jordan (2004) presented this methodology for stationary Gaussian time series, which complement the results obtained from the time domain. Moreover, sparse models deal directly with the limitations of complex high-frequency time series, such as complex structural and computational constraints.

In this paper, the main contribution was the description of a statistical methodological plot adopting the time domain series in the frequency domain combined with some dynamic spatial models, targeting a more in-depth understanding of an applied neuroscience research question. We demonstrated the validity and feasibility of this sequence of statistical approaches that could reveal a pattern toward brain activation, comparing the brain dynamic before and after a transcranial neuromodulation stimulation. The data were acquired following a systematic randomized controlled clinical trial protocol (Santos et al., 2018), using a sample of the EEG signals collected before applying high-definition transcranial direct current stimulation (HD-tDCS) over the temporal-parietal junction, under the polarity anode center condition and *post* the 2 mA current intensity in a single young healthy subject.

The motivation stems from the need to understand neuro-activation across different brain areas to analyze the effects of a focal transcranial brain stimulation and establish an innovative and effective neurorehabilitation strategy to treat verticality disorder after brain lesions (post-stroke). Moreover, the impact of this study will extend to the entire neuroscience/medical field that needs to adopt dynamic modeling for complex data; sparse models enable the use of big data demanding a low computational cost (shrinking the number of parameters in the model).

## 2. Methods

The paper is organized as follows. In subsection 2.1, we present an overview of the adopted experimental protocol. In subsection 2.2, we present the theoretical background for dynamic linear models, sparse estimation, sparsity in modeling, multilayer networks, network inference, and time series from a frequency-domain approach. In section 3, we discuss the empirical clinical results comparing different sparse estimations to distinguish patterns among different brain wavebands. Finally, some final comments are given in section 4.

### 2.1. Protocol Rational and Data Characterization

Neural systems' imbalance and degeneration related to postural control have led to new research regarding their origin and pathophysiology (Winter, 1995). In humans, different sensory information is used as pathways in the brain to maintain posture in the upright position (Day and Cole, 2002), and postural imbalance is one of the most common disorders after stroke. However, it has not been well-documented in the literature (Chern et al., 2010; Baggio et al., 2016). Hence, increasing knowledge about the effects of this strategy is essential for developing more effective rehabilitation protocols.

Non-invasive techniques of brain stimulation are current therapeutic resources related to the pathophysiology and behavior of the mechanisms that guide the human mind. Transcranial direct current electrical stimulation (tDCS) is a non-invasive neuromodulation technique that can model the cerebral function with a safe profile (Edwards et al., 2013). tDCS consists of electrodes unleashing weak electrical currents over the scalp, inducing cortical changes; it increases or decreases the local network excitability depending on the electrical current polarity.

At the neuronal level, tDCS affects polarization of the resting membrane potential, and this effect may acutely impact cortical excitability (Priori et al., 1998). Another effect may be related to the electrical dynamics of the neuronal membrane potential, as well as its change by at least 1 h (Nitsche et al., 2003). In addition, changes in the effectiveness of synaptic connections may last during the stimulation period. Studies on peripheral nerve and spinal cord stimulation have shown that direct current effects are also non-synaptic, with transient changes in the density of protein channels below the stimulation area (Ardolino et al., 2005; Cogiamanian et al., 2008). High definition tDCS (HD-tDCS) is a contemporary way of transcranial electrical stimulation, which promotes more focal stimulation than the conventional tDCS methods (please see Edwards et al., 2013).

In addition to these tDCS direct effects, “indirect” consequences come from connective-driven alterations of distant cortical and sub-cortical areas (Brunoni et al., 2012). Lang et al. (2005) revealed that stimulating the right frontopolar cortex (M1) with tDCS also activates several connected regions. Changes in brain activity, after the tDCS session, were also measured related to regions concerning blood flow using the sequential H1520 PET scan. In addition, by observing the stimulus area, the activation of “several motor areas” was observed, including “the caudal portion of the anterior cingulate cortex, cerebellum and superior temporal sulcus.” This could be due to a modulation of the functional interaction between M1 and these areas via cortico-cortical and cortico-subcortical connections.

Other studies using transcranial magnetic stimulation (TMS), also as a non-invasive neuromodulation technique, described the increased activity of the homologous area, contralateral to the stimuli (Siebner et al., 2000; Lee et al., 2003). Moreover, cerebral hemisphere interaction is commonly observed in the literature (Gilio et al., 2003; Plewnia et al., 2003).

These “indirect” changes on cerebral function are fundamental issues regarding the objective of the present study, which evaluated the effects of tDCS in the temporoparietal junction, the area related to postural control in humans (Winter, 1995). Inter-hemispheric interactions may contribute to defining the temporal and spatial features of voluntary movements, and consequently postural control (Meyer et al., 1998). There is a balance between these inter-hemispheric interactions, where each human cortex exerts inhibitory influences on the opposite motor cortex in normal conditions (Ferbert et al., 1992). Therefore, developing non-invasive techniques that modulate this balance will be a significant advance in the rehabilitation setting of stroke patients and other postural control disorders after more profound knowledge is gained of the technique's effects on the human brain.

**Figure 1:**
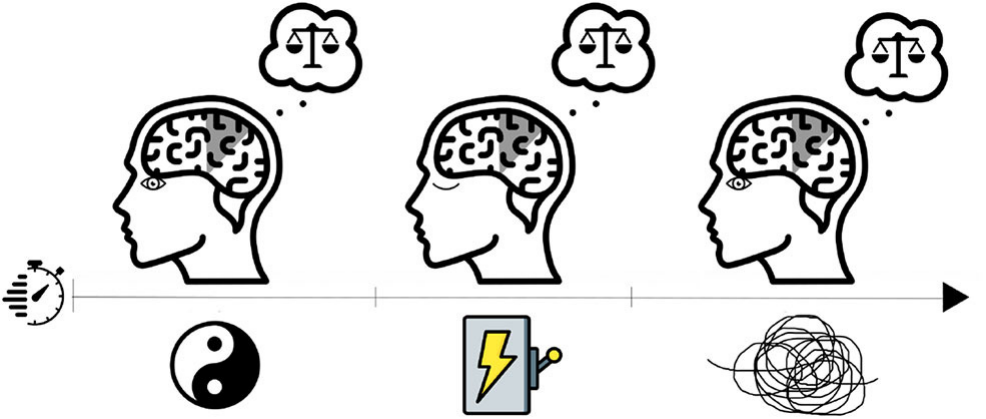
Visual representation of a resting-state baseline condition (left illustration; eyes open) in addition to stimulation stage (eyes closed) and accommodation post-stimulus (right illustration; eyes open). The main interest in the study is to compare the resting-state vs. accommodation post-stimulus.

**Figure 2:**
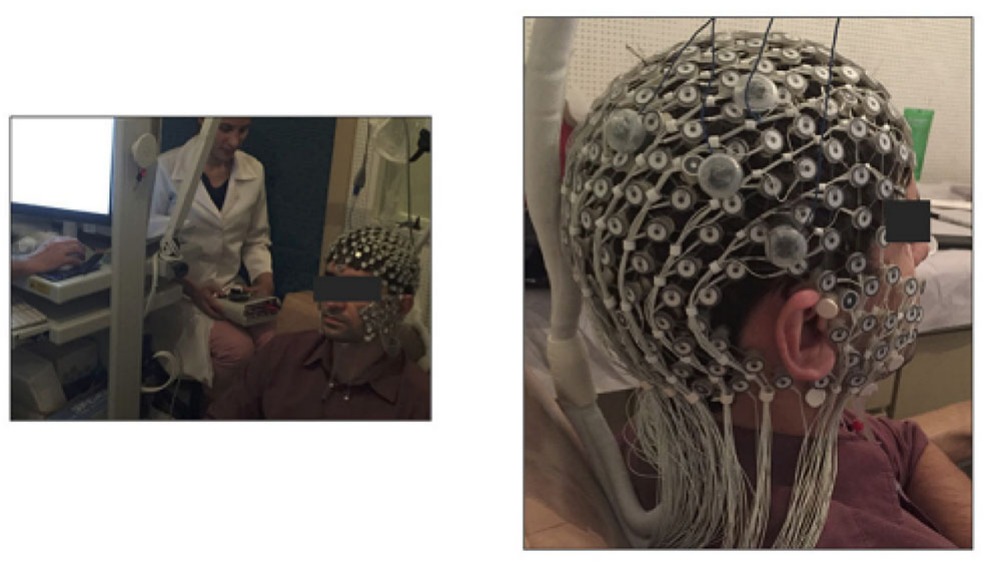
Photograph of the experimental trial (left-hand side) and the EEG cap (right-hand side) with small electrode array covering the scalp, while the large electrodes identifiable as a triangle configuration (four electrodes total) represent the tDCS stimulating electrodes (this image was previously published by Santos et al., 2018, under Open Access and Creative Commons Attribution License).

The current study was derived from a randomized double-blinded sham-controlled clinical trial that aimed to investigate a polarity and intensity-dependent shift in high-density EEG signals, following an intervention using high-definition transcranial direct current stimulation applied over the temporo-parietal junction in healthy subjects (Santos et al., 2018). The study protocol consisted of an HD-tDCS application over the right temporoparietal junction area, using a Soterix^Ⓡ^ NY-USA HD-tDCS with a constant current anode (active control). Four electrodes were used; the central electrode was placed over the circumcenter of P4-C4-T8 EEG coordinates, and the three peripheral electrodes were placed at a distance of 3 centimeters from the central electrode (over the EEG coordinates P4, C4, and T8). EEG recordings were made before and after each stimulation period, thus detecting ongoing changes in the raw EEG signals in response to tDCS (Figure 1). The total duration was 5 min of resting-state baseline condition added by 1.5 min of stimulation plus 5 min of accommodation post-stimulus, as shown in Figure 2 (for protocol details, please see Santos et al., 2018).

A dense array EEG signal was acquired using a 256-channel sensor net from Electrical Geodesics Inc. during the aforementioned electrical stimulation conditions. All channels were referenced to the vertex with reduced electrical impedance. The EEG was recorded continuously before and after the stimulation, excluding ramp-up and ramp-down periods (1.5 min total). The full trial experimentation lasted ~120 min. Previously, we discussed (Nascimento et al., 2019) some variations toward the Cathodal against the Active Control (Anodal) at the 2 mA condition; in this work we aimed to discuss an innovative statistical analyses of only one sample of the protocol experimentation compared to its reference (baseline).

Thus, in the present study, we analyzed and discussed the data set of a single healthy adult male participant during the resting state (baseline condition) and 45 s after an electrical stimulation. Each period (before and after stimulation) contains 5 min of observation, whereas the EEG sample rate was 500 Hz (500 observations per second), representing a total of 300,000 observations.

### 2.2. The Model

**Figure 3:**
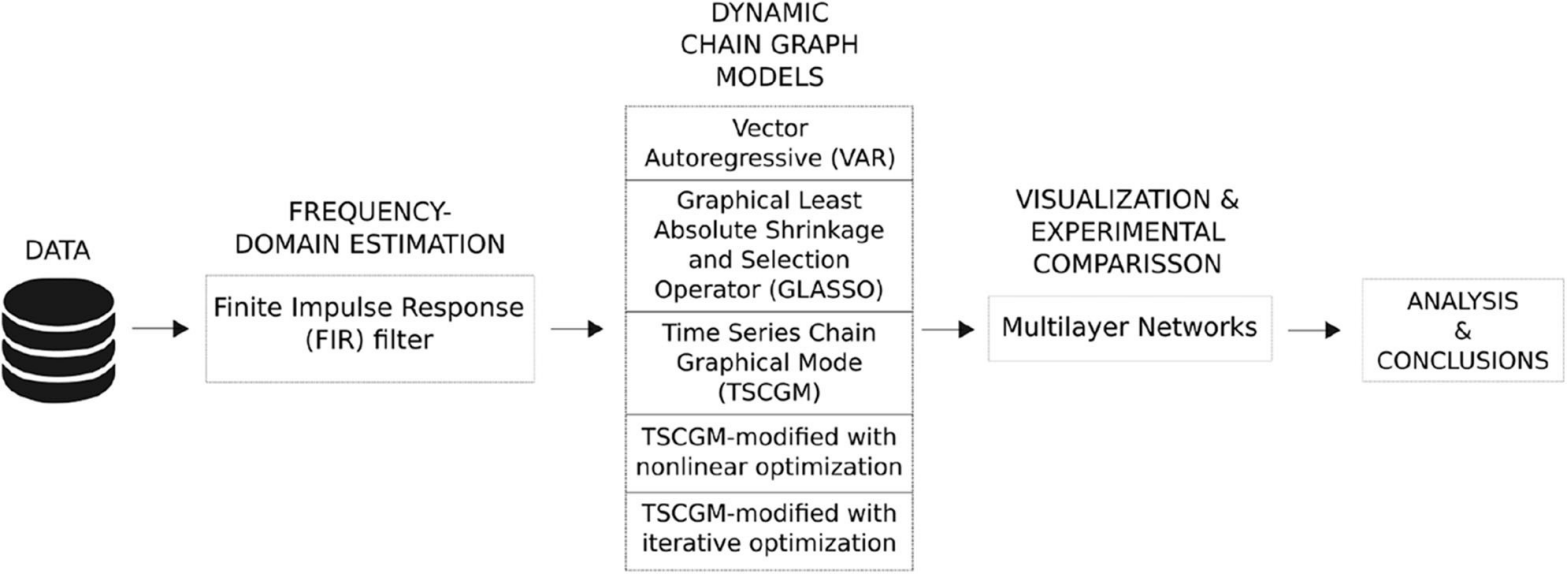
Visual summary of the methodological framework. Only one participant/trial was selected, and its biosignals were extracted using EEG during baseline period and 2 mA post-stimulation in order to compare the brain dynamic responses. As a pre-processing phase, an FIR filter was applied in the raw EEG signal aiming to estimate the brain frequency-domain phases. Then, five types of dynamic chain graph models were tested and compared, for instance, illustrated only with the filtered EEG alpha band. Later, all the bandpower were compared, given the outperformed model comparing resting-state vs. port-stimulation electrical brain dynamic.

Dynamic structure modeling may be considered as an alternative to estimate brain connectivity; additionally, it is natural to aggregate its estimated parameters into a graphical representation. Nonetheless, the dynamic model class is overparametrized (West et al., 1985; West and Harrison, 1989), especially in the time-varying approach, demanding some shrinkage of the parameter space (i.e., by adding sparsity to the parameter vector estimation process). A word of caution must be mentioned here; search patterns in small dimensions may deal with great noise (Nakao, 2016), added by limitations toward how to generalize the low-dimensional reduction approach (Rodrigues et al., 2016) and, for instance, brainwaves present a highly active process which comes with much noise (Natarajan et al., 2004). Therefore, filtering preprocessing is suggested to break the observed/raw time series signal into the frequency domain and then using the finite impulse response (FIR) filter. These elements are presented next and visually summarized in Figure 3.

#### 2.2.1. Dynamic Linear Model

The state space model is a flexible learning linear/non-linear dynamical system. As a particular case, the state transition and observation functions, known as a Dynamic Factor Model (DFM), may be expressed as a Gaussian linear process, often called a Dynamic linear model (DLM). For instance, consider a p-dimensional State Vector and m-dimensional observations, both normally distributed. At the initial time, (*t* = 0) presents the mean μ_0_ and variance $\sigma_{0}^{2}$,

$$\begin{array}{l} \theta_{0}\sim N_{p}\left(\mu_{0},\sigma_{0}^{2}\right) \end{array}$$

then for the time *t* ≥ 1,

$$\begin{array}{ll} \underset{\text{observation equation}}{\underset{︸}{Y_{t}=F_{t}\theta_{t}+\upsilon_{t}}}, & \;\upsilon_{t}\sim N_{m}\left(0,V_{t}\right),\\ \underset{\text{state equation}}{\underset{︸}{\theta_{t}=G_{t}\theta_{t-1}+\omega_{t}}}, & \;\omega_{t}\sim N_{p}\left(0,W_{t}\right) \end{array}$$

where matrices *G*_*t*_ (dimension *p* × *p*) and *F*_*t*_ (*m* × *p*) are known, followed by independent Gaussian random vectors υ_*t*_ and ω_*t*_ with mean equal to zero and known variance matrices *V*_*t*_ and *W*_*t*_.

Considering an ℝ^*p*^-valued and ℝ^*m*^-valued time series, we have the following: (i) (θ_*t*_) is a Markov chain and (ii) the observed time series (*Y*_*t*_), conditioned to (θ_*t*_). They are independent among the other time series and depends only on the associated state (θ_*t*_).

Moreover, this class of models is flexible given the possibility of incorporating more complex structures (locally they are linear, but globally perform as non-linear dynamic), by allowing the time-varying parameters, that is, compounding a latent variable in the estimation process. The estimation toward the state vector uses the conditional density π(θ_*k*_ ∣ *Y*), where *t* = 1, ..., *T* and *Y* are the observed values. Furthermore, *k* represents the recursive period and *t* the current period, where estimation problems are filtering (*k* = *t*), smoothing (*k* < *t*), and state prediction (*k* > *t*).

Filtering is a procedure that aims to update the current estimates as new data are observed π(θ_*t*_ ∣ *Y*_1:*t*_). Smoothing is a retrospective analysis, already containing all the observations in the series, which computes the conditional distribution θ represented by π(θ_*t*_ ∣ *Y*_1:*T*_), starting from π(θ_*T*_ ∣ *Y*_1:*T*_) back to front. Prediction is a forecast procedure that estimates the next observation based on the data π(θ_*t*+1_ ∣ *Y*_1:*t*_). Further details on Bayesian Forecasting and Dynamic models can be found in West and Harrison (1989) and Petris et al. (2009).

In contrast, the Vector Autoregressive (VAR) model is widely used in the literature (Krystal et al., 1999; Prado et al., 2006; Schlögl and Supp, 2006; Garrison et al., 2015) and recognize the non-linear dependencies between different brain regions, although may present limitations toward the curse of dimensionality. It is possible to impose restrictions on a VAR to make it “similar” to a factor model, i.e., such as DFM.

#### 2.2.2. Sparse Estimation Framework

Recent discoveries, related to time series modeling, discuss the challenge of estimating the model's dependency order, that is, related to the measure of complexity to high-dimensionality resolution. For instance, it enables the eigenvalues and eigenvectors to rotate in the state-space parameter dimension, given restrictions in the parameter vector space imposing some parameters to be equal to zero. Therefore, the main question may concern identifying the “best” and “simplest” approximation (without losing relevant information) that corresponds to the dynamic process.

This definition of “the best” is non-trivial given the lack of knowledge regarding the joint function related to the data and parameter associated with the phenomenon under study. The only available information is from the observed data as an information base in the estimation process. Several inferential methods may be adopted; among them, the most popular are maximum likelihood and ordinary least squares.

The sparse approach is equivalent to creating a bias toward sparsity in the maximum likelihood estimator (MLE), which may reduce the minimum square error. Thus, it sets conditions in the least squares aiming to minimize the *l*_1_-norm producing sparsity in the parameter vector θ. Additionally, prior knowledge can be incorporated, targeting only a subset of the parameter vector; to minimize a specific parameterization (θ_0_) problem, then

$$\begin{array}{l} \underset{s}{min}||\theta_{0}+s{||}_{1} \end{array}$$

truncating a NP hard problem (Chickering, 1996) into a linear programming (LP) problem in standard form (Zeemering, 2015). In general terms, adding vectorial assumptions concerning the reparatrization of the model associated with the parameter vector θ will impact the adjustment of the model and will be represented as an error vector [(*e*(θ)), which can be calculated according to a criterion, for example, least squares] that depends only on θ.

The search space is limited by models, some of them equivalent, which produce the same error vector value and least squares error (Tibshirani et al., 2012). That is, shrinkage may be applied through a singular value decomposition (SVD) to the matrix, which associates the number of constraints kernel of the Jacobian (*J*(θ)) or Hessian (*H*(θ)) matrices.

The non-linear least squares minimization method search direction (*s*(θ)) to refine the parameters by successive iterations may be adopted, such as a Newton method, described as

$$\begin{array}{l} s\left(\theta \right)=-\alpha H{\left(\theta \right)}^{-1}J{\left(\theta \right)}^{\prime }e\left(\theta \right). \end{array}$$

Based on the SVD results, values that assume a value equal to zero can be determined, thus setting a threshold if needed. A word of caution regarding the threshold; low values may bound the search space (then exclude valid directions to search for sparsity) and high values may change the model's behavior.

In contrast, other solutions may be obtained by the dual or primal linear programming (LP) problem. Deviation toward the search direction accuracy during the optimization procedure, through setting up a threshold, determines the quality of the maximization procedure. An application in the medical field, Zeemering (2015) used regression and state space classes of models in order to add sparse estimation to atrial fibrillation research.

The models adopted were classical VAR, Graphical Least Absolute Shrinkage and Selection Operator (GLASSO), Time Series Chain Graphical Model (TSCGM) and TSCGM-modified using Non-linear optimization over log-likelihood and Iterative optimizing the log-likelihood. The modified TSCGM, adopted in this work, considered an optimization option that uses the proportion of parameters equal to zero in relation to the total number of parameters of the model with a bias toward sparsity, in the MLE, whose minimization will occur through the *l*_1_-norm of the parameter vector and the Smoothly Clipped Absolute Deviation (SCAD).

#### 2.2.3. Sparsity in Modeling

The classical method for estimating connectivity matrices often uses the Vector Autoregressive (VAR) Model, which is a particular case of DLM when the parameters are invariant in time. For instance, consider a vector of observed variables *Y*, where *I* is an identity matrix, Matrices *X* represent *Y* lagged dependence, Γ_*j*_ are autoregressive parameters, and *u* is the error vector with covariance matrix Σ, using an ordinary least squares (OLS) standard estimation procedure equation by equation. Its vectorized form would be expressed as

$$\begin{array}{l} vec\left(Y\right)=\left(I_{m}⊗X\right)\Gamma +vec\left(u\right),\;where\;vec\left(u\right)\sim N\left(0,\Sigma ⊗I_{t}\right) \end{array}$$

where the matrix of coefficients Γ presents *m* × [# lagged variables + 1] dimension, which is the dynamic connectivity (also called effective connectivity), and the matrix of coefficients Σ represents the functional connectivity, where *t* represents the length of the *Y* series. The OLS estimation process can be translated by

$$\begin{array}{l} \text{log-likelihood}\left(\hat{\Gamma },\hat{\Sigma }|\text{observed data}\right)=\\ \underset{\Gamma ,\Sigma }{argmin}\left[\frac{1}{t}tr\left(\left(Y-X\Gamma \right)\Sigma^{-1}{\left(Y-X\Gamma \right)}^{\prime }\right)-log|\Sigma^{-1}|\right]. \end{array}$$

However, as the graph model also includes small linear dependencies, implying a number of larger links, it results in an exponential increase in relation to the number of channels, jointly impacting the interpretation of complexity and the processing/interpretation of results. Therefore, it is usual to use a data-dependent threshold to remove the weak connections, but selecting an appropriate value can be different according to the experiment setting and goals (Garrison et al., 2015).

An alternative approach is to reduce the number of links during the connectivity matrix estimation, using sparse time series models. One widely adopted model is the GLASSO, used as a sparse VAR and proposed by Friedman et al. (2008); the method takes into account the sparsity toward the estimation on the functional connectivity. Inherently, the estimated connectivity matrices often have few links, but, despite maximizing the likelihood of the observed biosignals regarding the proposed theoretical model, they can lead to a distinct dynamic/effective connectivity estimation.

For instance, consider *N* multivariate normal observations of dimension *p*, with mean μ, and covariance Σ. Using the empirical covariance matrix, the problem is to penalize the negative log likelihood,

$$\begin{array}{l} \text{ log-likelihood}(\hat{\Gamma },\hat{\Sigma }|\text{observed data})=\\ \underset{\Gamma ,\Sigma }{argmin}[\frac{1}{t}tr((Y-X\Gamma )\Sigma^{-1}(Y-X\Gamma {)}^{\prime })-log|\Sigma^{-1}|+\\ \;\lambda_{1}\sum_{i=1}^{G}\|\gamma_{i}{\|}_{2}+\lambda_{2}\sum_{k\neq k^{\prime }}\left\|\Sigma_{kk^{\prime }}^{-1}\right\|] \end{array}$$

with λ_1_ and λ_2_ penalty parameters, γ_*i*_ is a subvector of Γ, *G* = *q*^2^ total number of groups and *k* block coordinate descent derived from Σ (that is, shrinking only in part of the covariance matrix).

A generalization of this model is found in the TSCGM, proposed by Abegaz and Wit (2013), where sparse estimations of both effective and functional connectivity matrices are obtained. In this method, both matrices are estimated interactively: first, a sparse functional connectivity estimate is calculated with a non-sparse non-concave penalty (smoothly clipped absolute deviation, SCAD); and, later, sparse effective connectivity using the previous estimation as an initial value. This cycle is performed until it reaches convergence. For further details, please see Abegaz and Wit (2013).

TSCGM has been successfully applied to genetic data, and when applied to electroencephalograms, numerical experiments have shown a considerable reduction in the number of estimated connections. However, TSCGM also distorts the strength of some links, creating connections that were not present using a VAR model, because it relies on GLASSO to estimate the functional connectivity in each iteration.

The approach behind TSCGM is remarkable for increasing the sparsity of the estimations. Since the algorithmic implementation presented some issues during its application with biosignals, we introduced some adjustments. We also used a TSCGM-modified model that estimates the effective and functional connectivity that maximizes the loglikelihood of the model simultaneously using a Newton-type numerical optimization method. These methods are the non-linear optimization and iterative optimization. For more in-depth discussions toward sparsity profile, please see Benson et al. (2003), Wipf and Nagarajan (2008), and Rakotomamonjy (2011).

#### 2.2.4. Multilayer Networks

Graph models are useful for describing and exploring patterns of dynamic/effective and functional/contemporaneous interactions of a given phenomenon. In human neuroscience experimentation, brain network connectivity activation can be recorded from the electrical impulse aiming to highlight interaction among areas.

Given the complexity of the brain, multilayer networks incorporate the multivariate and multi-scale information scheme (De Domenico, 2017). In general, multilayer networks can be seen as a collection of several distinct classic networks, which separately encode a specific type of information about the system as a layer, thus composing a multilayer network at the end. Those layers quantify some elements of similarities, such as (i) activity in different frequency bands, (ii) time-varying activity, (iii) activity of different tasks, and (iv) structural and functional connectivity.

Alongside this information, two important concepts about brain networks are essential; first the *functional connectivity*, which expresses the statistical correlation within a time step, also interpreted as contemporaneous interactions, and the second concept is related to *effective connectivity* in which it describes the dynamics of the current time in relation to previous times (this is the dynamics of the present response in relation to the lagged responses) (Friston, 2011).

#### 2.2.5. Inferential Network Analyses

Let us start discussing the concept of conditional independence. It should be mentioned that part of this subsection was inspired by Højsgaard et al. (2012). Consider a collection of random variables (*X*_ν_) ν ∈ *V* associated along with a joint density, where V is a finite node set. Now, let us arbitrarily select three subsets of V (suppose A, B, and C); *X*_*A*_ = (*X*_ν_) ν ∈ *A* as well as for *X*_*B*_ and *X*_*C*_. The statement *X*_*A*_ and *X*_*B*_ is said to be conditionally independent given *X*_*C*_ (that is, *A* ⫫ *B* ∣ *C*) if for each observation *x*_*C*_ of *X*_*C*_, *X*_*A*_, and *X*_*B*_ are independent in the conditional distribution given *X*_*C*_ = *x*_*c*_. In this context, a generic probability function, π( ), defines the characterization *A* ⫫ *B* ∣ *C* as

$$\begin{array}{l} \pi \left(x_{A},x_{B}|x_{C}\right)=\pi \left(x_{A}|x_{C}\right)\pi \left(x_{B}|x_{C}\right), \end{array}$$

and rewriting as two functions *g*( ) and *h*( ), then

(1) $$\begin{array}{l} \pi \left(x_{A},x_{B},x_{C}\right)=g\left(x_{A},x_{C}\right)h\left(x_{B},x_{C}\right). \end{array}$$

Whenever possible to describe the joint density as a product of functions, as in Equation 1, adopting the conditional independence approach, this is known as the factorization criterion. Hence (*X*_ν_) ν ∈ *V* can be represented as a set of joint densities, for instance, described as a parametric model, enabling us to use the factorization form, adopting the conditional independence relations between the variables. Often described as an undirected graph, conditional independence models unravel patterns out of a complex application. Suppose that G=(V,E) is an undirected graph with cliques (maximal complete subset) *C*_1_, …, *C*_*k*_. The factorization form occurs if the joint density π() of the variables in V is

$$\begin{array}{l} \pi \left(x_{\nu }\right)=\overset{k}{\underset{i=1}{\prod }}\left(g_{i}\left(x_{C_{i}}\right)\right) \end{array}$$

where functions *g*_1_()…*g*_*k*_() depend on x only through *x*_*Cj*_ according to the condition that π() factorizes according to G.

The global Markov property ensures that through the model it factorizes in all densities given G, then G encodes the model's structure through the conditional independence; that is, whenever sets are separated by another in the graph, it is said that conditional independence happens under the model.

Nevertheless, there is not a unique equivalence/representation corresponding to patterns of conditional independences represented by a chain graph G, guarded by the Markov properties. A chain graph is a combination of no bidirected edges and no semi-directed cycle graphs and may be seen as a natural generalization of undirected graphs and directed graphs that is acyclic (DAG).

For instance, the Markov properties can be described as two-step factorization; the first step represents the joint density as sub-parts; similar to a DAG, the search for the separation that maximizes the information is described as a graph form.

$$\begin{array}{l} \pi \left(x_{V}\right)=\underset{C\in C}{\prod }\pi \left(x_{C}|x_{pa\left(C\right)}\right) \end{array}$$

where C is the set of components of G. Each conditional density π(*x*_*C*_ ∣ *x*_*pa*(*C*)_) will be based according to an undirected constructed graph;

That is, the form of subgraph G is induced by *C* ∪ pa(C), disregarding the directions, in relation to all possible pa (C). A hierarchy should be considered since some variable sets *pa*(ν) ν ∈ *V*, in relation to the variables in pa(ν) precede v. It is worth mentioning that the vertices of the graph represent the random variables, enabling us to identify the sets pa(ν) with the parents (descendent) of ν in the DAG.

Let us consider a chain graph (or complex network) for a given network defined by a set of vertices *V* and a set of edges *E* order in pairs, then each point is represented as G=(V,E). The interpretation of edges (also called links) can be also dynamic, as they are indexed in time, which represents the evolution of the interaction between pairs of vertices.

Time series data modeling can combine dynamic graphical models, which enables us to incorporate sparsity, aiming to estimate statistical causality and correlation across series. For the sake of simplicity, let us consider Markovian dynamics (time *t* relates only to time *t* − 1), which are similar to VAR(1), as

$$\begin{array}{l} \left(a,\;b\right)\in V_{t}\times V_{t-1}\Leftrightarrow \Gamma_{ab}\neq 0 \end{array}$$

where effective connectivity is represented by the link between area *a* and *b* at consecutive time steps related to an element from Γ (points across time). Similarly, functional connectivity is represented by the estimated links associated with the effects corresponding to the precision matrix Σ (correlation within the same time period); this is related to the models' errors as

$$\begin{array}{l} \left(a,\;b\right)\in V_{t}\times V_{t}\Leftrightarrow \Sigma_{ab}\neq 0. \end{array}$$

Thus, a multivariate time series can be translated into a learning probabilistic connection network structure (as a graph model), aiming to estimate brain connectivity networks. The Dynamic Chain Graph Model (DCGM) creates a multivariate dynamic linear model for each chain component, and Wermuth and Lauritzen (1990) discuss the class of dynamic graphical models that enables us to estimate different signal phases and compare their structural relations. For instance, the dynamic/contemporaneous interactions between brain regions, presented by Costa et al. (2017), as a particular case of its theory in the neuroscience field.

#### 2.2.6. TS Frequency Domain Approach

Brain activity can be collected as biosignals, composing the information flow from a group of connected neurons (called a neural circuit). These activities may seem at first to be pure noise, but between specific ranges, they may distinguish hidden patterns (Prado and West, 2010; Scheffer-Teixeira et al., 2013). Moreover, different frequency bands can contribute toward the brain mapping functionality by maximizing the information flow through the brain regions (according to the observed and latent components).

The literature presents changes in the frequency cuts (Fransson, 2005; Su et al., 2013), and those hubs might be very different when measured at different frequency bands. The findings concern the topological information measured from components at different frequencies (in hertz unit—Hz). Thus, such an enriched representation (decomposed TS signal) is more valuable than other aggregated representations (raw TS signal). For instance, some pass band ripple filters are Butterworth, Chebyshev, Elliptic or Cauer, and Finite Impulse Response (FIR) filter (for further details, please see Parks and Burrus, 1987).

Moreover, results presented in the literature (Newson and Thiagarajan, 2018; Wojcik et al., 2018) suggest that a healthy human brain operates at a transition point between independent and highly dependent frequency bands (e.g., represented as functional layers). EEG raw signals enable us to establish encoding the connectivity between the neural circuit, and are described within five frequency bands. It is reasonable to adopt the delimitation of biosignals in frequency bands theta (0.01–4 Hz), delta (4–8 Hz), alpha (8–16 Hz), beta (16–32 Hz), and gamma (32–49 Hz).

De Domenico (2017) suggests that brain activity may be represented in functional layers, without acting independently between them, adopting existing mechanisms for integration and segregation across different frequency bands. Thus, adopting multilayer techniques is shown to be potential in biomarkers as it integrates the whole concept of interdependence and is applicable in neurological and mental studies.

Thus, this work adopted the finite impulse response (FIR) filter, used to filter the limit of the signal coefficients given some order and frequency cutoff. Additionally, we added a correction using a Forward and Reverse filter applied to the FIR obtained signal to correct the phase distortion introduced by a one-pass filter, although this approach exerts a magnitude in the process in which it is equivalent to square responses. Both tools are implemented in R (Octave Forge, 2007), presented in the package signal.

The multiplex sparse dynamic model framework enables us to map the network connections, across different layers encoded as frequency bands (although integrated as De Domenico, 2017 suggests). Furthermore, the irreducibility of the multilayer functional representation of the human brain increases the need for multilayer analysis of the underlying architecture, targeting the identification of hubs.

## 3. Results

Neuroscientists have attempted to understand brain connectivity through the functional and effective connectivity among brain areas, using biosignals, such as Electroencephalogram (EEG) or functional Magnetic Resonance Imaging (fMRI). This work tried to fathom the brain manipulation task related to the perception of verticality and posturography as a novelty targeting the development of a therapeutic approach for post-stroke patients.

A previous study performed the recording of high-density EEG together with the evaluation of visual vertical (VV). The authors mapped the high-density evoked potential with the evoked potential analysis discriminating the location of brain activation during VV evaluation. The authors verified brain activity during the task with a focus on the right lateral tempo-occipital cortex (Lopez et al., 2011). These physiological findings reaffirm the hypothesis of the dominance of the right cerebral hemisphere in the control of vertical perception. They also highlighted the right temporoparietal junction (TPJ) as a key point in the judgment of vertical orientation (Dieterich et al., 2003; Karnath and Dieterich, 2006; Pérennou et al., 2008; Baier et al., 2012).

Our group developed a promising brain stimulation protocol applied on right TPJ using a bipolar mount with conventional transcranial direct current stimulation (tDCS) and right hemisphere high-definition tDCS (HD-tDCS). We verified the efficacy and safety of this protocol in VV manipulation in healthy individuals.

For instance, Santos and Edwards (2019) pointed out that investigations toward the influence of cortical activity using non-invasive electromagnetic brain stimulation (NIBS) suggests understanding and treating verticality disorders as a neurorehabilitation. Thereby, Santos et al. (2018) implemented a protocol toward human verticality manipulation, using neuromodulation, on healthy participants aiming to understand the recovery of this intentional artificial brain lesions, briefly introduced in section 2.1.

**Figure 4:**
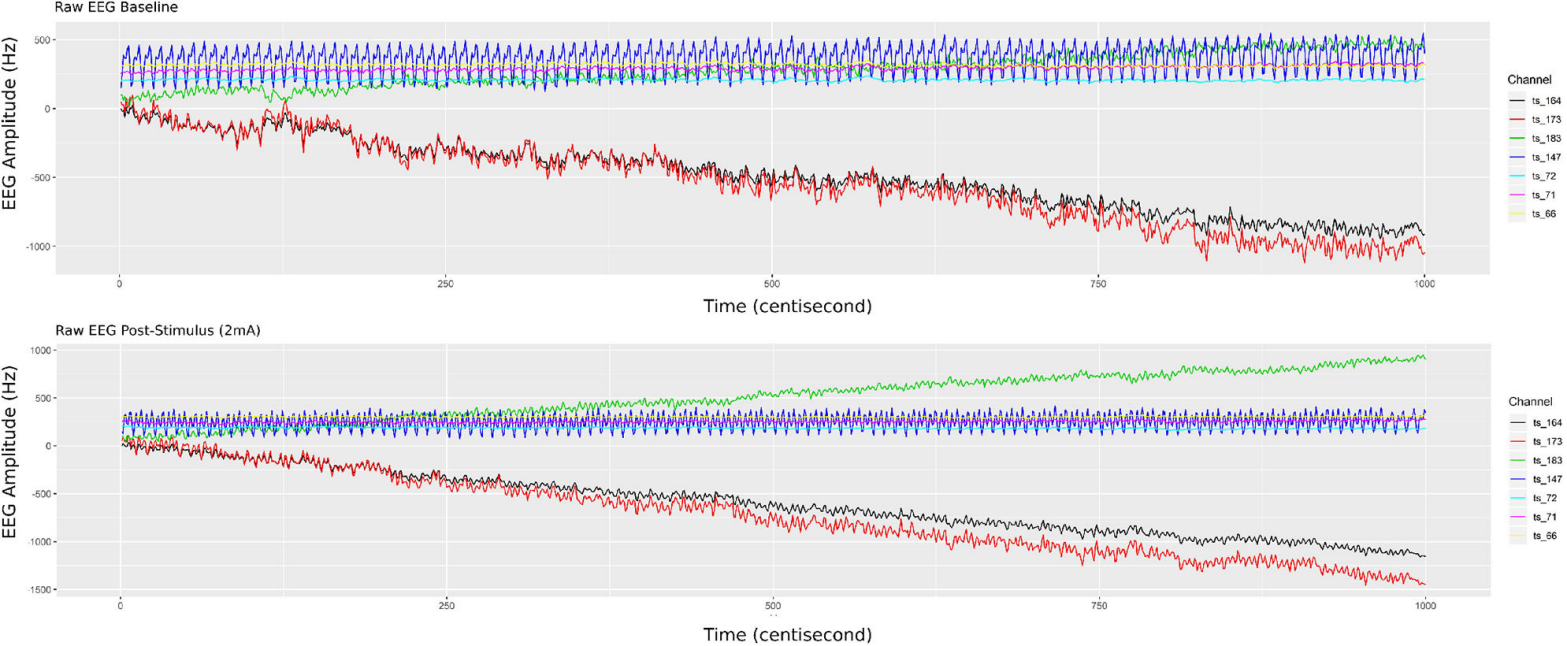
Single participant EEG raw signals from 5 min of recorded biosignals (top panel) during the resting state and (bottom panel) after HD-tDCS–Anodal Center 2 mA–stimulation. The brain responses' amplitude (on the y-axis), from the raw EEG signals, increased after the stimulation.

Randomly selecting a single participant, Figure 4 illustrates 5 min of brain response in each panel (raw EEG signals), selecting only seven channels (out of 256), and compares the signals during the resting state (top panel) vs. post-2 mA stimulation (bottom panel). Most of the selected EEG channels were located in the motor cortex; three channels were derived from the right hemisphere (164, 173, and 183) and located nearby the region placed the tDCS four electrodes. Then, three other channels were derived from the left hemisphere (66, 71, and 72) in which they are physiologically related to those selected from the right hemisphere; additionally the EEG channel 143 was placed in the parietal cortical region.

It can be observed in Figure 4 that post-stimulation of the brain response amplitude from the raw EEG signals increased, which is more related to the hemisphere side to channels 183, 164, and 173 (related with the tDCS placed region). In addition, channel 66 has had its signal shifted up, which is physiologically explicable due to the polarity dependence created by the applied stimulus (directly related to channel 164, through the anodal input current electrode). According to Ombao and Ho (2006), Prado and West (2010), and De Domenico (2017), studies provide traces that brain connectivity may be better understood using frequency band decomposition limiting the influence of noise in the brain signal and describing different brain tasks as oscillatory bands.

**Figure 5:**
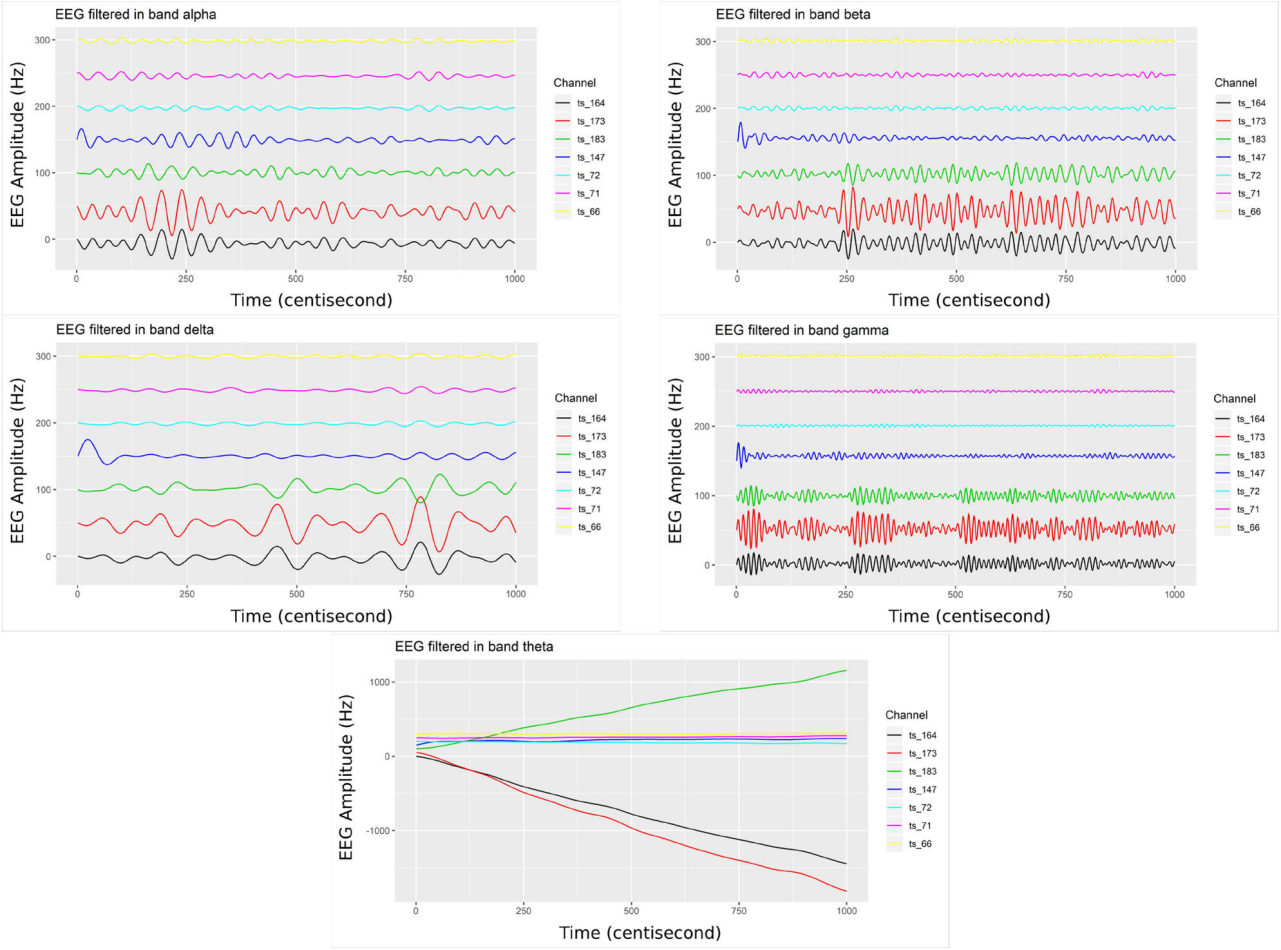
Bandpower from the filtered EEG signals (top left) considering the alpha band, (top right) filter in the beta band, (middle left) in the delta band, (middle right) in the gamma band, and (bottom center) filter in the theta band. The EEG electrodes placed on the right-side brain hemisphere present higher dynamic/variation (channels 164, 173, and 183), related to post-2 mA stimulation.

Initially, we filtered the raw EEG signals, adopting the FIR with pass-band filter, utilizing five fundamental bands of brain waves (alpha, beta, delta, gamma, and theta). Figure 5 shows only the filtered signals related to the post-stimulation period, whereas elucidating the difference in band oscillation (signal phases) for each channel.

The channels located on the same brain hemisphere side as the neuromodulation (tDCS), presented greater oscillation. Thus, this dynamic may be translated/associated with the electrical transferred activity (energetic dissipation). This activity is expected given the rise of entropy through electrical synergy in this area (Nascimento et al., 2019).

The study of the human brain has been developing and generates an enormous amount of data, however, revealing the information extracted from this complex system is not trivial and, often, aggregating this information may lead to erroneous results (Fiecas and Ombao, 2011; Castruccio et al., 2016; Shen et al., 2016). Alternatively, the multilayer network approach provides a mathematical background to model and analyze complex data with multivariate and multi-scale information (Kivelä et al., 2014). Multiplex network shapes can be formatted using (i) activity in different frequency bands, (ii) time-varying activity, (iii) activity with respect to different tasks, and (iv) structural and functional connectivity.

Thus, estimations regarding the representation of a joint distribution of random variables are needed (the network structure). This procedure seeks to describe the causal relations across the brain regions. The Vector Autoregression (VAR) model would be appropriate to describe a brain connectivity network, nonetheless, it may present a high curse of dimensionality in large sets. This class of models presents a significant number of parameters to be estimated. Additionally, shrinkage either in the data (such as PCA) or parameter spaces (like GLASSO and TSCGM) is not straightforward and may lead to misleading information.

The graphical LASSO (GLASSO) model, proposed by Friedman et al. (2008), estimates that matrices tended to be different from those determined by a classical VAR method. It was noticeable that non-sparse VAR estimation not only increased the sparsity of the effective connectivity matrix but also “created links” that did not appear before (based on our empirical analysis). These models present a high sensitivity to non-stationary series and might mislead the estimation point connections (given the shrinkage on the covariance matrix–Contemporaneous Effect–, thus changing the dynamic interactions).

**Figure 6:**
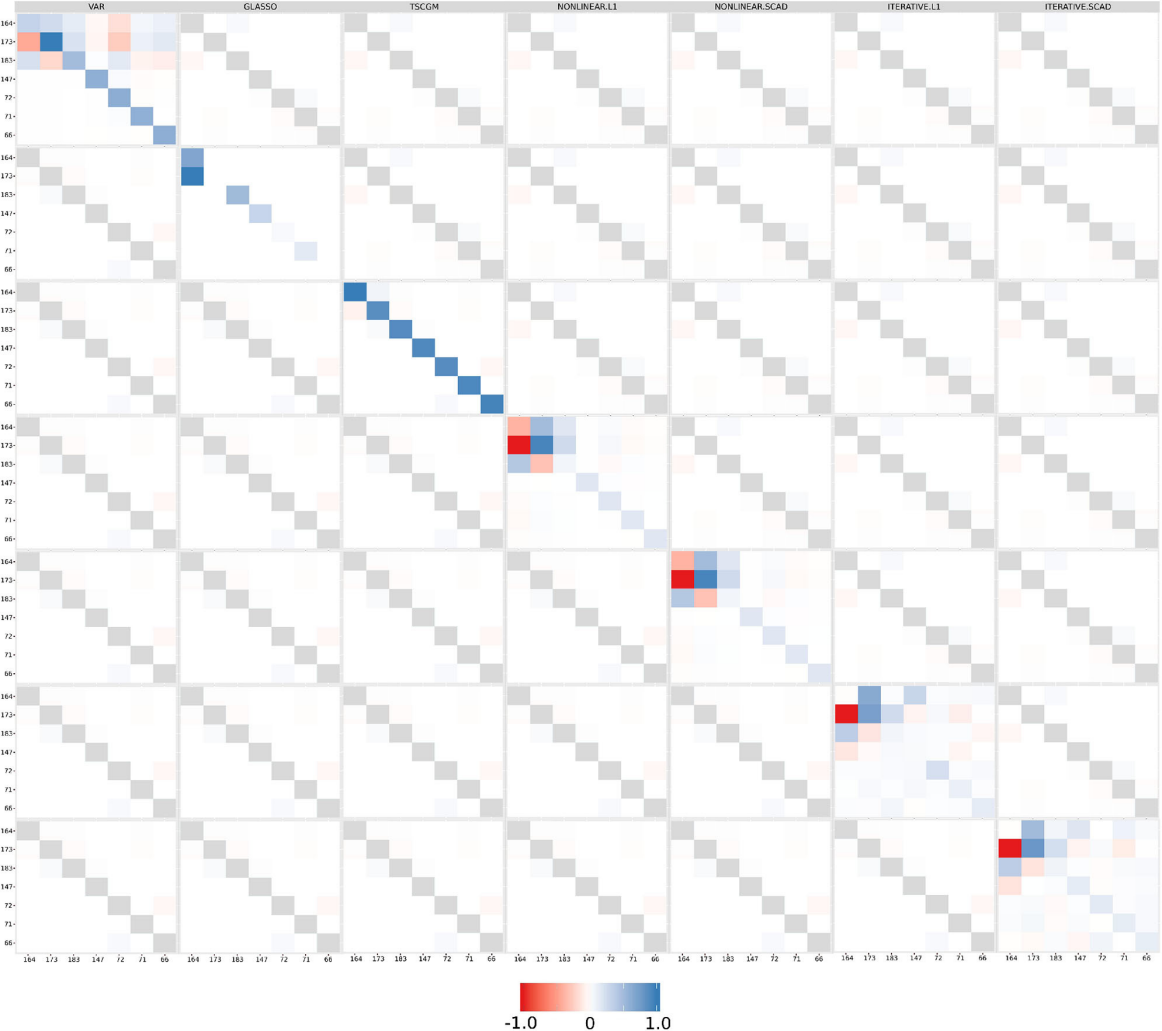
Functional connectivity as the supra-adjacency matrix in which rows and columns form groups from the seven filtered EEG alpha frequency-band signals, throughout the methods (VAR, GLASSO, TSCGM, TSCGM non-linear *l*_1_-norm and SCAD, and TSCGM-iterative *l*_1_-norm and SCAD). The VAR method is the reference, whereas the target is to maintain the strong links and remove the weak using sparsity. The TSCGM non-linear provided a competitive insight preserving the structure and function of the human brain.

Alternatively, TSCGM and TSCGM-modified was performed using a non-linear optimization over the log-likelihood, and iterative optimizing the log-likelihood (with *l*_1_-norm and SCAD penalization, not only in the covariance matrix) (Abegaz and Wit, 2013). Figure 6 shows the supra-adjacency matrix related with the functional connectivity, across seven EEG channels, comparing seven estimation methods (classic VAR, GLASSO, TSCGM, TSCGM non-linear *l*_1_-norm, TSCGM non-linear SCAD, TSCGM-iterative *l*_1_-norm, and TSCGM-iterative SCAD), for instance, only the performance of a single band (alpha).

The VAR model includes weak linear dependencies, as mentioned in section 2.2, and it is desirable to use a data-dependent threshold to remove the weak connections without losing information. GLASSO and TSCGM led to different interpretations, compared to the VAR-estimated matrix. Nevertheless, TSCGM-modified with non-linear optimization using both *l*_1_-norm and SCAD penalization maintained the strong links presented in the VAR but also eliminated the weak ones, therefore suggesting a competitive performance among the others. The same cannot be said for the TSCGMs-modified with iterative optimization.

**Figure 7:**
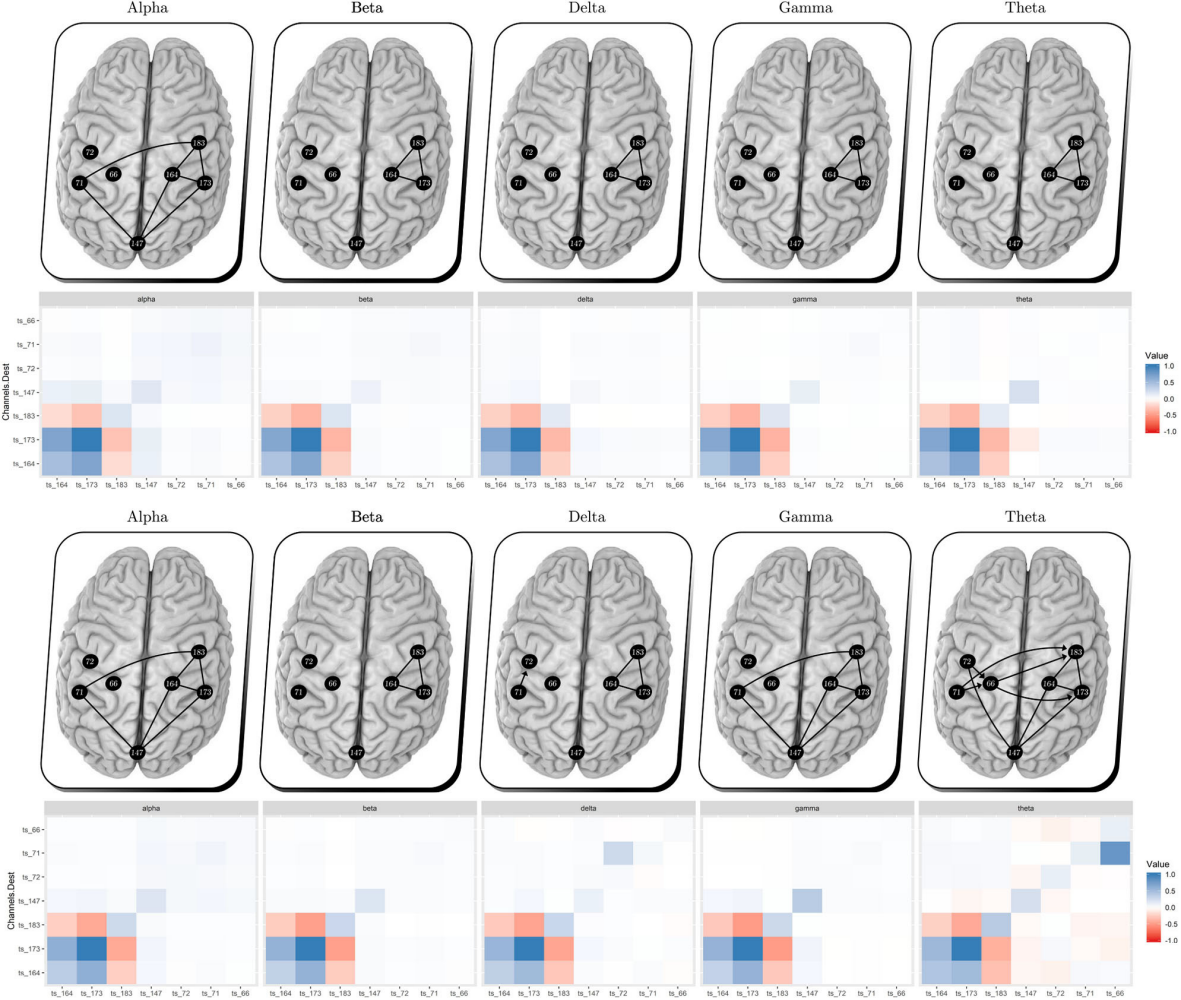
Multiplex EEG signals, per bandpower, (top panels) resting state and (bottom panels) after unilateral HD-tDCS–Anodal Center 2 mA–stimulation. Comparing the resting state vs. post-stimulation, we obtained additional links within the gamma and theta bands during post-stimulus, which suggests an outgrowth in the electrical brain dynamic.

Figure 7 shows the estimated brain dynamic/effective connectivity among the seven filtered channels (top figures) during the resting state and (bottom figures) post-stimulation, adopting the performance of TSCGM non-linear optimization using SCAD. That is, the brain illustrates with the correlation matrices the neuronal information floating connectivity (in different frequency-band signals).

No visual modification can be observed through the analysis of the alpha, beta, and delta bands, according to Figure 7. Gamma and theta bands show a slight change (considering the new estimated coefficient intensity during post-stimulus). In agreement with the present findings, previous results showed gamma band change after brain stimulation (Santos et al., 2018).

The results were similar to the findings observed in patients after stroke. Our data thus indicate that the proposed approach may be a promising tool for methodological-analysis toward the treatment of verticality error in stroke patients (Santos-Pontelli et al., 2016; Santos et al., 2018). In previous studies, Nascimento et al. (2019) compared HD-tDCS dose-response, adopting the same protocol study, which included the placebo/sham HD-tDCS trail response, that by its statistical results, it helped to validate the sparse dynamic models' feasibility effects search of HD-tDCS and its pure effect.

## 4. Final Remarks

This study aimed to implement and discuss the comparison of sparse methods toward parameter dimension shrinkage. Nevertheless, preserving information from empirical data is necessary to develop elements for brain manipulation intervention related to the perception of verticality and posturography as a novelty aimed at the recovery of post-stroke patients. The multilayer network approach enabled us to integrate the information retained given the electrical post-stimulus synergy (through different frequency bands).

The findings obtained in this paper contribute to the process of estimating the neuronal circuit connections, with robust inference and computational feasibility. Estimating a network structure can be a non-trivial (Chickering, 1996), highly complex task (Rodrigues et al., 2016), despite the fact that these sparse models showed to be promising, bypassing the false positives link estimation (results in Figure 6).

As demonstrated in the present work, the sparse models (using a dynamic linear model) combined with the frequency domain approach represented as the multilayer network implement to the neuroscience field the capability of interpreting/estimating the dynamic of the neural circuits based on EEG data in a comprehensive way. Moreover, we aimed to contribute with more in-depth data analysis toward the protocol (Santos et al., 2018), discussing its feasibility, enlightening the human manipulation intervention response dynamic.

This work is limited given that conclusions are based on a single participant response, whereas future works intend to extend this modeling using hierarchical models and interpretation of the entire sample and protocol. Liao et al. (2017) showed that the modular structures of brain networks completely vary across individuals. Thus, hierarchical modeling is required in the form of a set of state vectors for each chain component, as an exchangeable sample with the common mean. Therefore, future work shall explore the time-varying parameters, enclosed by the dynamic linear models, in a hierarchical version, suitable for interventions, such as those presented here, indexed in time.

## Data Availability Statement

The datasets generated for this study are available on request to the corresponding author.

## Ethics Statement

The studies involving human participants were reviewed and approved by Joao P. Leite—Department of Neuroscience and Behavioral Sciences, Ribeirao Preto Medical School, University of São Paulo. The patients/participants provided their written informed consent to participate in this study.

## Author Contributions

DN and MP-O: statistical analysis, computational modeling, interpretation of the data, and manuscript writing. JL: study concept, interpretation of the data, and critical revision of the manuscript. DE: study concept and design of the clinical trial, interpretation of computational modeling, data analysis, and critical revision of the manuscript. FL: supervision of the statistical analysis, interpretation of the data, and critical revision of the manuscript. TS: study concept and design of the clinical trial, project management, data acquisition, supervision of the data analysis, data interpretation, and manuscript writing. All authors contributed to the article and approved the submitted version.

## Conflict of Interest

The authors declare that the research was conducted in the absence of any commercial or financial relationships that could be construed as a potential conflict of interest.
